# Endoscopic Treatment of External Coxa Saltans (Snapping Hip) in the Supine Position

**DOI:** 10.1016/j.eats.2024.103328

**Published:** 2024-11-22

**Authors:** Tyler R. Mange, Kory B. Dylan Pasko, Dean Wang

**Affiliations:** Department of Orthopaedic Surgery, University of California at Irvine, Orange, California, U.S.A.

## Abstract

External coxa saltans, or “snapping hip,” is present in about 5% of the population and typically stems from a tight iliotibial band (ITB) passing over the greater trochanter. For patients who have been unresponsive to nonoperative treatments, endoscopic ITB release can be effective in relieving symptoms. Various techniques have been described, including those involving lateral decubitus positioning, alternative portal placement, and outside-in release of the ITB. In this technique, we describe an endoscopic partial ITB release for external coxa saltans using supine positioning, standard hip arthroscopy portals, and inside-out release from the peritrochanteric space, which allows treatment of concomitant intra-articular pathology and direct visualization of the posterior undersurface of the ITB for a more precise release of the involved pathologic tissue.

External coxa saltans, or “snapping hip,” is part of a larger collection of conditions known as greater trochanteric pain syndrome.^1^^,^^2^ External snapping hip can lead to lateral-sided hip pain and reduced hip range of motion. The condition results from a thickened area of the iliotibial band (ITB), usually involving the posterior aspect of the ITB, that snaps over the greater trochanter (GT) with hip flexion. This can result in secondary inflammation of the GT bursa, causing pain.^1^, ^2^, ^3^ Patients with this condition also may have intra-articular pathology that can contribute to symptoms around the hip.^4^^,^^5^ Symptomatic cases of external coxa saltans can sometimes be alleviated with nonoperative treatments, including a combination of rest, stretching and physical therapy, and anti-inflammatory modalities including corticosteroid injection of the GT bursa.^1^, ^2^, ^3^

When nonsurgical treatment fails, multiple surgical techniques to address external coxa saltans have been described. These include various open techniques such as Z-lengthening or release of the ITB, as well as endoscopic techniques that may result in a lower risk of wound complications compared with open approaches.^1^^,^^3^^,^^6^, ^7^, ^8^ Many of the currently described endoscopic techniques are performed with the patient in the lateral position, which may preclude simultaneous treatment of intra-articular conditions such as a labral tear or impingement.^1^^,^^7^, ^8^, ^9^ The use of accessory portals also results in deviation from the standard hip arthroscopy technique for many hip arthroscopy surgeons. In this technical note, we describe an endoscopic technique for ITB release for the treatment of external coxa saltans in the supine position on a standard hip arthroscopy traction table, allowing for concomitant treatment of intra-articular pathology. Furthermore, through standard hip arthroscopy portals (anterolateral, mid-anterior, and distal anterolateral [DALA]), this technique is performed from the peritrochanteric space, which allows for direct visualization of the posterior thickening of the ITB that is commonly involved in the pathology (Table 1, Video 1).

**Table 1:** Pearls and Pitfalls of the Described Surgical Technique

Pearls	Pitfalls
Establish visualization in the peritrochanteric space by gently sweeping with a blunt trochar and resecting bursa with a shaver, similar to establishing visualization in the subacromial space in the shoulder.	Ensure traction is released and the leg is slightly abducted before entering the peritrochanteric space.
Fluoroscopy can also be used to confirm proper positioning of the arthroscope.	Avoid complete anterior-to-posterior release or over-resection of the ITB.
After ITB release, the hip should be taken through a full range of motion to confirm resolution of mechanical snapping.	With the patient in a supine position and viewing through the midanterior portal using a 70° arthroscope, the arthroscope needs to be suspended with the surgeon’s arm. Otherwise, the arthroscope is prone to fall posteriorly in the peritrochanteric space.

ITB, iliotibial band.

### Preoperative Workup

Patients presenting to the clinic with painful snapping hip are evaluated with routine history and physical examination as well as imaging of the affected hip(s) that includes standard anteroposterior views of the hips and pelvis, Dunn lateral, and false-profile radiographs and magnetic resonance imaging. If the patient has not responded to a satisfactory trial of nonoperative management, endoscopic surgery is offered, and the patient is counseled on the risks, benefits, and postoperative course. If concomitant intra-articular pathology is diagnosed and possibly symptomatic, additional workup that includes computed tomography and diagnostic intra-articular or peritrochanteric injection may be performed to ensure that all symptomatic pathology is addressed during surgery.

## Surgical Technique

**Fig 1:**
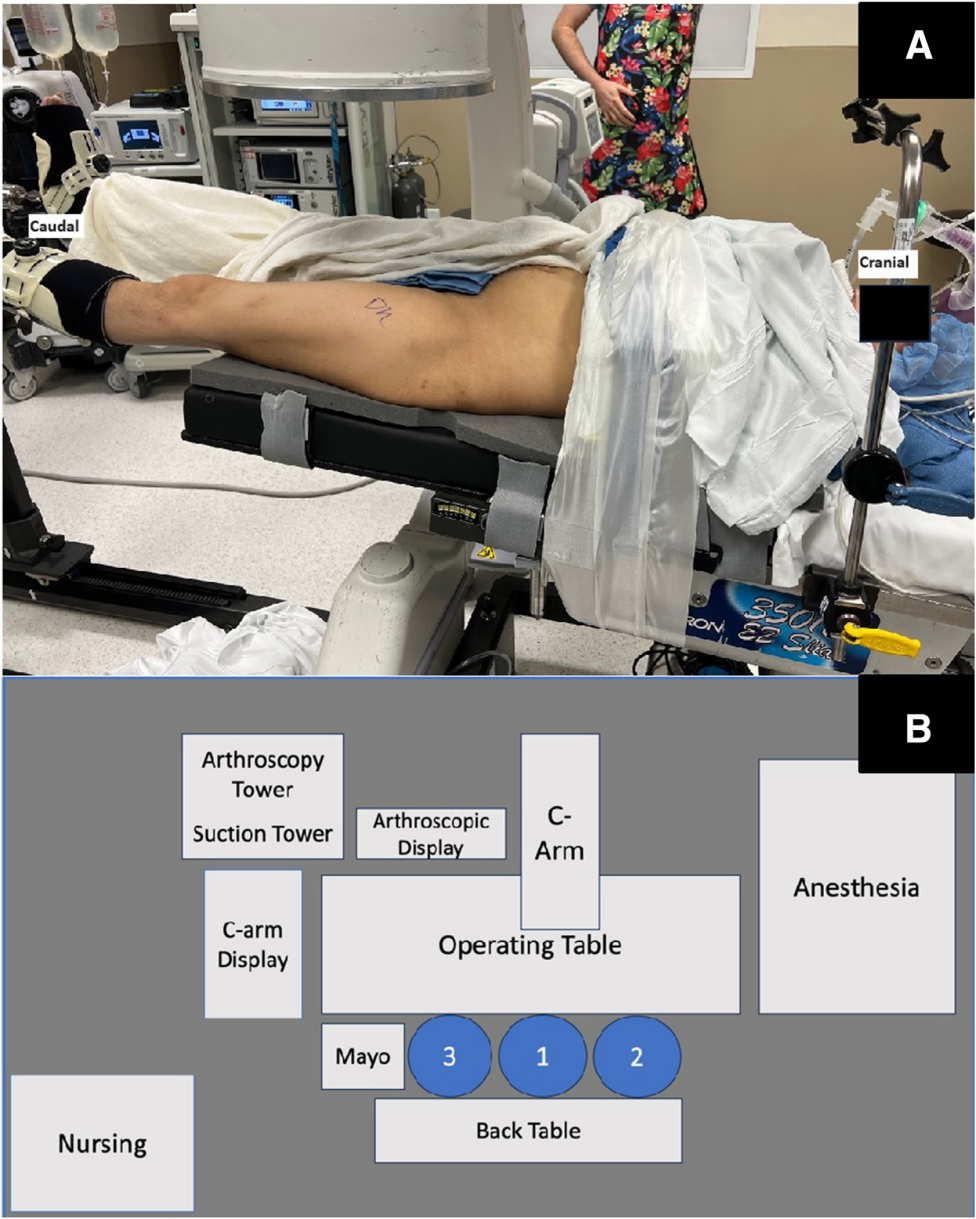
(A) Layout of operating room with patient positioned supine on traction table for a left hip procedure. (B) Schematic of operating room layout for left hip procedure (1: surgeon, 2: assist, 3: surgical tech).

**Fig 2:**
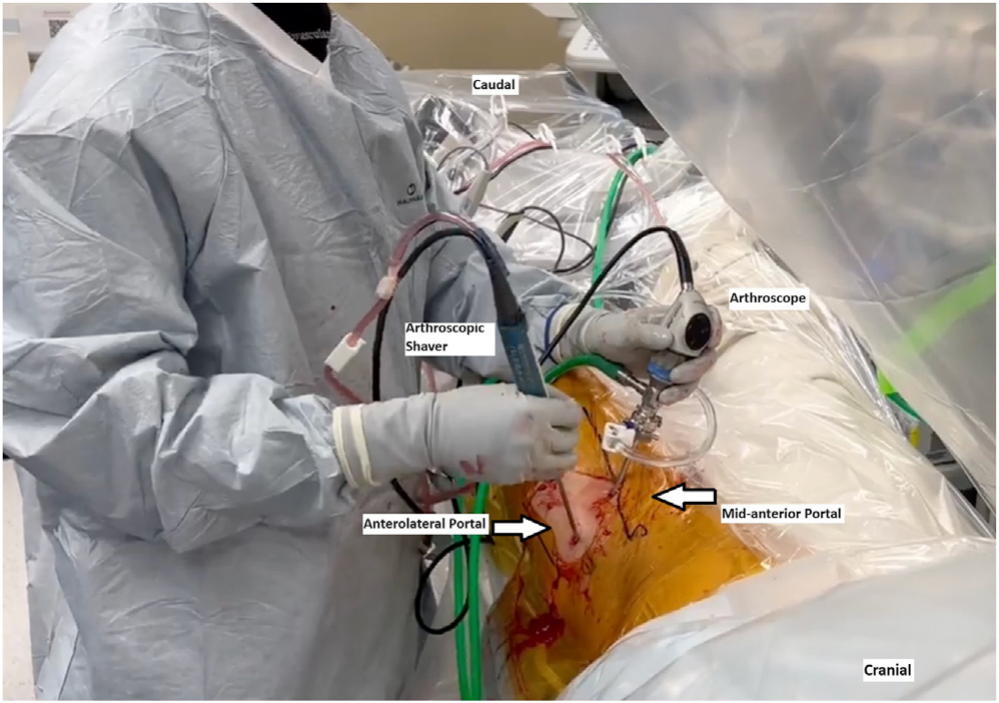
In a left hip, the patient is positioned supine with the 70° arthroscope placed through the midanterior portal, and the arthroscopic shaver is placed through the anterolateral portal for resection of the trochanteric bursa and visualization of the undersurface of the iliotibial band and surrounding structures.

**Fig 3:**
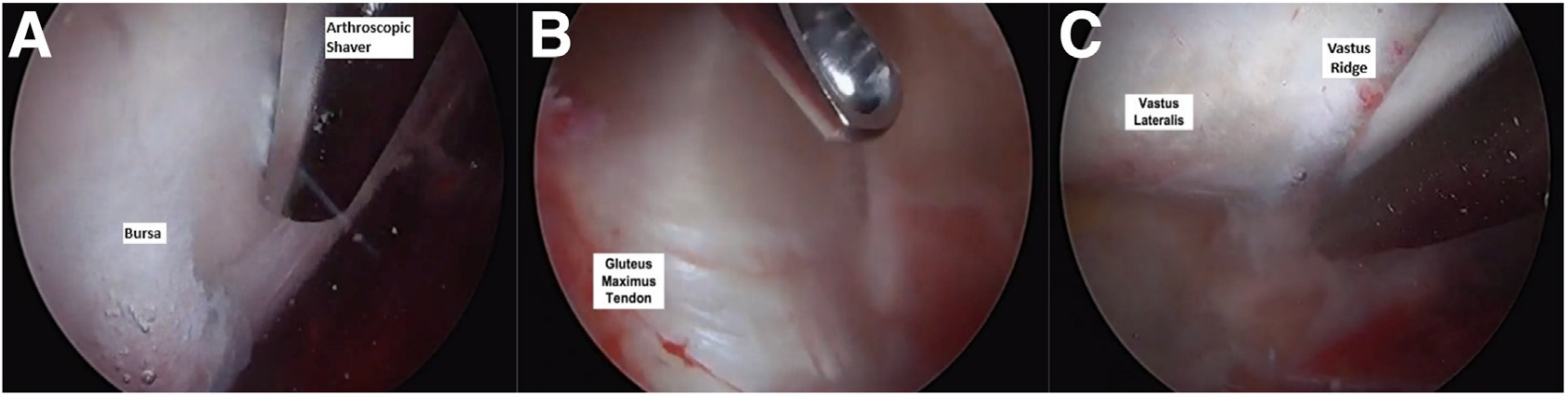
In a left hip, the patient is positioned supine with the 70° arthroscope placed through the midanterior portal. (A) Resection of the bursa over the vastus ridge. (B) Visualization of the gluteus maximus tendon insertion on the proximal femur. (C) Visualization of the vastus lateralis following bursal resection.

The patient is positioned supine on a postless hip arthroscopy traction table (Fig 1). An examination under anesthesia may be useful to identify the provocative maneuvers that results in the snapping hip. After the intra-articular portion of the hip arthroscopy is completed, traction is released, and the surgical leg is abducted 10° to 20°. The foot is typically kept internally rotated, matching the degree of femoral version to position the femoral neck parallel to the floor. Through the midanterior portal, the blunt trochar and cannula are inserted into the peritrochanteric space through the interval between the sartorius and tensor fascia lata. The blunt trochar and cannula are swept proximal and distally in the peritrochanteric space to sweep away the bursa and establish a window for visualization, similar to the technique used to establish visualization in the subacromial space in the shoulder. The 70° arthroscope is then inserted through the cannula and the inflow opened. An arthroscopic shaver can then be introduced via the DALA and/or anterolateral portal to resect the trochanteric bursa (Fig 2). Enough bursa should be resected to allow for adequate visualization of the undersurface of the ITB, gluteus maximus insertion on the proximal femur, vastus lateralis and vastus ridge, and abductor tendon insertions on the GT (Fig 3). The deep posterior surface of the ITB is often thickened and erythematous in the external snapping hip.

**Fig 4:**
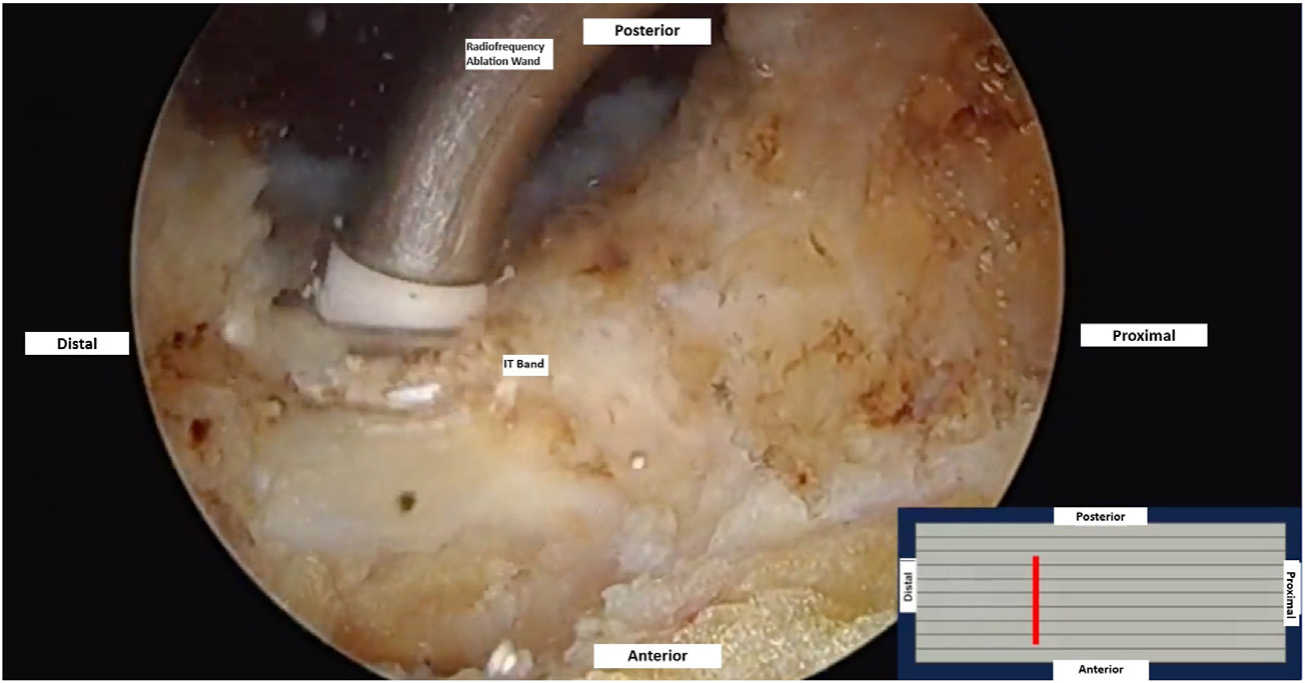
In a left hip, the patient is positioned supine with the 70° arthroscope placed through the midanterior portal. Endoscopic view shows anteroposterior release of the posterior thickening of the undersurface of the ITB using a radiofrequency ablation wand. This portion of the ITB is commonly the offending area that snaps over the greater trochanter. (ITB, iliotibial band.)

**Fig 5:**
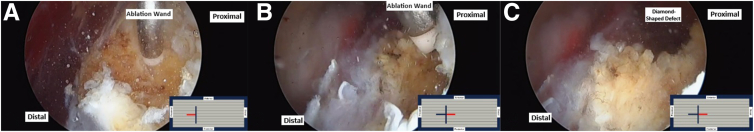
In a left hip, the patient is positioned supine with the 70° arthroscope placed through the midanterior portal. (A) Endoscopic view shows the extension of the ITB release distally through the thickened posterior tissue. (B) Endoscopic view shows the extension of the ITB release proximally through the thickened posterior tissue. (C) Endoscopic view shows the resultant diamond-shaped defect after debridement of the triangular flaps from the cruciate release. (ITB, iliotibial band.)

Working through the anterolateral and/or DALA portal, a cruciate release is then made within the offending portion of the posterior ITB that snaps over the GT. Using a radiofrequency ablation wand, the anterior-to-posterior portion of the cruciate incision is made first (Fig 4). Care is taken to leave intact ITB fibers anteriorly and posteriorly to avoid a complete release. Proximal and inferior limbs are then created centered over the posterior thickening of the ITB. The 4 resultant flaps are then resected with a shaver, leaving a diamond-shaped defect (Fig 5). All fluid is then evacuated out of the space with suction, and the hip can then be manually brought through flexion and extension to check for resolution of snapping. If residual snapping is appreciated, additional resection of the offending tissue can be performed. Standard closure of the portals is then performed. Immediately after surgery, the patient is encouraged to begin full passive range of motion, stretching exercises, and lateral scar massages to prevent excessive scar tissue formation at the site of the ITB release.

## Discussion

In summary, we describe an endoscopic inside-out ITB release for external coxa saltans using standard hip arthroscopy portals and supine positioning. One benefit of this technique is that it allows for direct visualization of the offending portion of the ITB through the peritrochanteric space. The ability to directly visualize and accurately target the problematic ITB tissue with endoscopy allows for a precise release and reduces the risk of incomplete treatment, complete ITB transection, or iatrogenic damage to surrounding structures.

Concomitant hip pathologies such as labral tears, chondral lesions, and/or impingement can exist in patients with external coxa saltans and also can contribute to their hip pain and dysfunction.^10^ Although the diagnosis of external coxa saltans often may be straightforward, determining whether concomitant intra-articular pathologies are causing symptoms can sometimes be more obscure. If they are symptomatic, addressing only the ITB may result in persistent hip pain after surgery. Use of this described technique allows the surgeon to perform a diagnostic arthroscopy and treat the concomitant pathology as needed without the need to make additional portals, modify patient positioning intraoperatively, or stage the surgical procedures. The use of standard hip arthroscopy portals and supine positioning with this technique aligns with the established protocols of most hip arthroscopy surgeons, facilitating ease of adoption in clinical practice. This streamlined approach contributes to a safer, more efficient, and reproducible operation. Table 2 summarizes the advantages and disadvantages of the presented technique.

**Table 2:** Advantages and Disadvantages of Endoscopic Treatment of External Snapping Hip Using Traditional Hip Arthroscopy Portals and Supine Positioning

Advantages	Disadvantages
Supine positioning on a standard traction table allows for seamless transition between intra-articular hip arthroscopy and peritrochanteric ITB release.	With the patient in a supine position and viewing through the midanterior portal using a 70° arthroscope, additional technical skill is required.
Use of standard hip arthroscopy portals aligns with the established protocols of most hip arthroscopy surgeons, facilitating ease of adoption into clinical practice.	Traction boots must be appropriately padded and applied to avoid soft-tissue complications on the foot and ankle. The boots can be loosened during the endoscopic portion of the case.
Avoids additional portals and intraoperative patient repositioning.	

ITB, iliotibial band.

## Disclosures

The authors declare the following financial interests/personal relationships which may be considered as potential competing interests: D.W. reports consulting or advisory for Newclip Technics, DePuy Synthes Mitek Sports Medicine; consulting or advisory and funding grants from Vericel; travel reimbursement from Arthrex; consulting or advisory and equity or stocks from Cartilage; equity or stocks from Overture Orthopaedics; and funding grants from. All other authors (T.R.M., K.B.D.P.) declare that they have no known competing financial interests or personal relationships that could have appeared to influence the work reported in this paper.

## Supplementary Data

Video 1 After positioning the patient supine on the postless traction table, a diagnostic arthroscopy of the left hip with a 70° arthroscope is performed via midanterior and anterolateral portals. A distal anterolateral portal can be established if needed. After intra-articular pathology is addressed as indicated, traction is released, and the peritrochanteric space is established by first placing the blunt trochar in the midanterior portal and through the interval between the sartorius and tensor fascia lata. The space is established via blunt dissection followed by use of the arthroscopic shaver to resect the bursa until adequate visualization is achieved. From the peritrochanteric space, the offending area of the ITB is then identified, and a cruciate release is performed with a radiofrequency ablation wand. The resulting triangular flaps are then resected with an arthroscopic shaver, leaving a diamond-shaped defect in the ITB. The hip can then be ranged fully to ensure complete resolution of the snapping. (ITB, iliotibial band.)

## References

[1] Malinowski K., Kalinowski Ł., Góralczyk A., Ribas M., Lund B., Hermanowicz K. (2020). External snapping hip syndrome endoscopic treatment: “Fan-like” technique as a stepwise, tailor-made solution. Arthrosc Tech.

[2] Redmond J.M., Chen A.W., Domb B.G. (2016). Greater trochanteric pain syndrome. J Am Acad Orthop Surg.

[3] Allen W.C., Cope R. (1995). Coxa saltans: The snapping hip revisited. J Am Acad Orthop Surg.

[4] Domb B.G., Curley A.J. (2023). Editorial Commentary: Treatment of concomitant intra-articular pathology in patients with greater trochanteric pain syndrome is indicated by provocative impingement or instability physical examination and ultrasound-guided analgesic injection testing. Arthroscopy.

[5] Yee C., Wong M., Cohen D. (2023). Labral tears and chondral lesions are common comorbidities identified during endoscopic repair of gluteal tendon tears for greater trochanteric pain syndrome: A systematic review. Arthroscopy.

[6] Voos J.E., Rudzki J.R., Shindle M.K., Martin H., Kelly B.T. (2007). Arthroscopic anatomy and surgical techniques for peritrochanteric space disorders in the hip. Arthroscopy.

[7] Ilizaliturri V.M., Martinez-Escalante F.A., Chaidez P.A., Camacho-Galindo J. (2006). Endoscopic iliotibial band release for external snapping hip syndrome. Arthroscopy.

[8] Randelli F., Mazzoleni M.G., Fioruzzi A., Giai Via A., Calvisi V., Ayeni O.R. (2021). Surgical interventions for external snapping hip syndrome. Knee Surg Sports Traumatol Arthrosc.

[9] Mak C.Y., Lui T.H. (2022). Endoscopic treatment of recurred external snapping hip after endoscopic iliotibial band release. Arthrosc Tech.

[10] Maldonado D.R., Blein R.M., Lee M.S. (2022). Patients with concomitant painful external snapping hip and femoroacetabular impingement syndromes reported complete snapping resolution with release of the gluteus maximus and iliotibial band, and comparable minimum 2-year outcomes to a propensity-matched control group. Arthroscopy.

